# Comparative Evaluation of Inoculation of Urine Samples with the Copan WASP and BD Kiestra InoqulA Instruments

**DOI:** 10.1128/JCM.01718-15

**Published:** 2016-01-28

**Authors:** Jesper Iversen, Gitta Stendal, Cecilie M. Gerdes, Christian H. Meyer, Christian Østergaard Andersen, Niels Frimodt-Møller

**Affiliations:** Department of Clinical Microbiology, Hvidovre Hospital, Copenhagen, Denmark

## Abstract

This study evaluated the quantitative results from and quality of the inoculation patterns of urine specimens produced by two automated instruments, the Copan WASP and the BD InoqulA. Five hundred twenty-six urine samples submitted in 10-ml canisters containing boric acid were processed within 30 min on an InoqulA instrument plating 10 μl of specimen, and on two WASP instruments, one plating 1 μl of specimen (WASP-1), and the second plating 10 μl of WASP (WASP-10). All samples were incubated, analyzed, and digitally imaged using the BD Kiestra total lab automation system. The results were evaluated using a quantitative protocol and assessed for the presence or absence of ≥5 distinct colonies. Separate studies were conducted using quality control (QC) organisms to determine the relative accuracy of WASP-1, WASP-10, and InoqulA instruments compared to the results obtained with a calibrated pipette. The results with QC organisms were calculated as the ratios of the counts of the automated instruments divided by the counts for the calibrated pipette (the gold standard method). The ratios for the InoqulA instrument were closest to 1.0, with the smallest standard deviations indicating that compared to a calibrated pipette, the InoqulA results were more accurate than those with the WASP instrument. For clinical samples, the WASP instruments produced higher colony counts and more commensals than the InoqulA instrument, with differences most notable for WASP-1. The InoqulA instrument was significantly better at dispersing organisms with counts of ≥10^5^ bacteria/ml of urine than were the WASP-1 and WASP-10 instruments (*P* < 0.05). Our results suggest that the InoqulA quantitative results are more accurate than the WASP results, and, moreover, the number of isolated colonies produced by the InoqulA instrument was significantly greater than that produced by the WASP instrument.

## INTRODUCTION

Recent years have seen the introduction of new instrumentation in the microbiology laboratory (1, 2), and it is the opinion of experts that this trend will accelerate in the near future (1). Despite the availability of plating and streaking instruments from different manufacturers, there is a scarcity of studies comparing the actual relative performances of the instruments.

In this study, we compared the performances of two front-end plating and streaking instruments, the Copan WASP (Copan, Brescia, Italy) and the BD InoqulA (BD Kiestra B.V., Drachten, the Netherlands), for use with plating clinical urine specimens. For both instruments, fully automated plating and streaking can be performed with liquid-based specimens only, although both instruments can streak specimens that have been manually inoculated. The WASP instrument uses calibrated loops of various sizes to both inoculate and streak liquid-based specimens. The InoqulA uses a disposable pipette tip to dispense liquid-based specimens onto plates and patented magnetic bead technology to spread the specimen over the agar surface.

In our laboratory, we utilized 2 WASP instruments and 1 InoqulA instrument to plate clinical specimens so we could use instruments that our technicians routinely utilize.

The minimum volume that the InoqulA instrument can plate is 10 μl, and, to our knowledge, this is the volume plated for urine specimens by most InoqulA users. This volume is also recommended for urine cultures, for which the criterion of ≥10^3^ bacteria/ml of urine is used to define significant bacteriuria (3). The WASP instrument can plate a minimum of 1 μl of urine, and we are aware of some users who plate 1 μl with the WASP, while other users plate 10 μl. Consequently, in this study, we compared the relative performances of the InoqulA instrument plating 10 μl of urine and the WASP plating 1 μl and 10 μl of urine.

## MATERIALS AND METHODS

### Quantitative comparison of WASP, InoqulA, and calibrated pipette.

In order to evaluate the accuracy of the quantitative inocula of the 1-μl and 10-μl loops of the WASP instrument (WASP-1 and WASP-10, respectively) and the 10-μl automatic pipette of the InoqulA instrument (InoqulA-10), we produced suspensions of two control strains, Escherichia coli ATCC 25922 and Enterococcus faecalis ATCC 29212, at 10^3^ and 10^4^ CFU/ml in sterile saline and ran them on the instruments. As a comparison, quantitative subcultures were performed in parallel with a 100-μl calibrated pipette, changing the pipette tip for each subculture; this is considered the gold standard method. The suspensions were produced by harvesting colonies from an overnight agar plate culture and mixing in sterile saline to a 0.5 McFarland standard with ∼10^8^ CFU/ml and diluting to an estimated 10^4^ and 10^3^ CFU/ml. Each subculture/inoculation was plated on three 5% blood agar plates and the quantitative counts calculated as the mean count of the three plates after 18 h of incubation at 35°C. Quantitative counts of the different subcultures were compared by relating the CFU per milliliter of the automated instruments with the CFU per milliliter of the calibrated pipette as a ratio, i.e., the CFU per milliliter of the automated inoculum divided by the CFU per milliliter of the calibrated pipette counts for the same suspension. This experiment was repeated four times for each inoculum and each control strain, i.e., 2 × 2 × 4 ratios.

For the WASP and InoqulA instruments, the following manufacturer-installed streaking patterns were used for the experiments: WASP-1, “single streak type 5”; WASP-10, “single streak type 5”; and InoqulA-10, “07 zigzag 3,5-1 s200.” These were the same streaking patterns used in the study with routine urine cultures and can be seen in Fig. 2.

### Testing of clinical specimens.

This study utilized 526 consecutive urine samples submitted by primary care physicians in 10-ml Sarstedt Urine Monovette tubes with boric acid. Each urine sample was processed within a 30-min time period on the WASP-1, WASP-10, and InoqulA-10 instruments. The order of inoculation was randomized. With the WASP-10 and InoqulA-10, 10 μl of preserved specimen was plated, and with the WASP-1, 1 μl of preserved specimen was plated on chromogenic agar (Brilliance UTI-agar; Oxoid) and 5% horse blood agar. All inoculated plates were loaded onto the BD Kiestra total lab automation (TLA) system and incubated at 35°C for 18 h (in ambient air), at which time the plates were individually digitally imaged utilizing the standard BD plate imaging system and software.

All digital images were evaluated by the study investigators. A predetermined scheme was developed to standardize the quantification of pathogens and contaminants and to determine the presence of discrete colonies. Adequate isolation of a particular colony morphology was defined as ≥5 isolated colonies. In our experience, 5 isolated colonies is a sufficient amount to perform a diagnostic workup, e.g., matrix-assisted laser desorption ionization–time of flight mass spectrometry (MALDI-TOF MS) and antimicrobial susceptibility testing.

The plates were read according to routine Hvidovre Hospital (Copenhagen, Denmark) microbiology laboratory protocols, i.e., typical urinary tract pathogens (e.g., E. coli, Klebsiella spp., Proteus spp., Citrobacter spp., Enterobacter spp., Enterococcus faecalis, and Staphylococcus saprophyticus) were quantified if ≤2 urinary tract pathogens were present in the culture. The presence of >2 urinary pathogens was classified as a mixed culture. Typical urinary tract commensals (e.g., coagulase-negative staphylococci) were recorded as such.

### Statistical methods.

The ratios calculated from the manual pipette and the automated instrument experiments were expected to be nonsignificantly different from 1.0, which was tested by a paired *t* test. A comparison of the frequencies was performed with a chi-square test or Fisher's exact test, according to a number of samples above or below 50, respectively. A *P* value of <0.05 was considered significant.

### Colony count standards.

Colony count controls were prepared with 10-fold serial dilutions to serve as the template for growth for 10^2^, 10^3^, 10^4^, and 10^5^ CFU/ml for each of the 3 instruments. Actual colony counts were performed on the 10^3^ and 10^4^ dilutions to verify that the colony counts were as intended. Digital images were captured for the calibrated pipette method and the WASP-1, WASP-10, and InoqulA-10 instruments, and these images were used as educational aids for assessing all colony counts for growth on plates in this study.

## RESULTS

### Comparative colony counts.

The results of the preliminary experiments that were performed to compare the relative colony counts of the E. coli and E. faecalis suspensions obtained with the InoqulA-10, WASP-1, and WASP-10 instruments with the quantities obtained with a calibrated 100-μl manual pipette are presented in Table 1 as the ratios of the CFU per milliliter of the WASP-1, WASP-10, and InoqulA-10 instruments compared to those obtained with the gold standard, the calibrated pipette. All means were significantly different from 1.0, except for InoqulA-10 and WASP-10 for the inoculum with 10^3^ CFU/ml of E. coli. However, the mean ratios were lowest and had the smallest standard deviations for InoqulA-10 for both concentrations for E. coli and E. faecalis (Table 1), indicating that the InoqulA inoculation instrument was the most precise. The mean WASP-1 ratios were 0.5 - to 1-log higher than the standard, with mean ratios of around 5 to 12 times higher than those for both control strains.

**TABLE 1:** Ratio of quantitative results for InoqulA-10 and WASP-1 and -10, as related to results obtained with a calibrated pipette^*a*^

Data by series	Mean ratios by instrument					
	10^3^ CFU/ml of urine			10^4^ CFU/ml of urine		
	InoqulA-10	WASP-10	WASP-1	InoqulA-10	WASP-10	WASP-1
Series A: E. coli						
Experiment						
1	1.36	0.43	24.03	1.55	1.83	3.85
2	1.27	1.44	6.34	1.25	1.50	4.44
3	0.94	1.65	2.88	1.17	1.36	4.36
4	0.84	4.46	14.46	1.18	2.60	6.84
Mean (SD)	1.10 (0.25)	2.00 (1.73)	11.93 (9.42)	1.29 (0.18)	1.82 (0.55)	4.87 (0.34)
*P* vs 1.00^*b*^	NS^*c*^	NS	*	*	*	*
Series B: E. faecalis						
Experiment						
1	1.69	1.69	9.46	1.18	1.59	5.37
2	1.25	1.69	4.03	1.37	1.83	3.85
3	1.17	1.94	3.88	1.41	1.69	5.17
4	1.97	2.90	14.75	1.32	2.88	13.15
Mean (SD)	1.52 (0.38)	2.06 (0.57)	8.03 (5.18)	1.32 (0.10)	2.00 (0.60)	6.89 (4.24)
*P* vs 1.00^*b*^	*	*	*	*	*	*

a Shown are the mean ratios of 3 experiments for each combination of inoculator and bacterial species for intended quantities of 10^4^ and 10^3^ CFU/ml of urine. The means of calibrated pipette results are normalized to 1.0.

b *P* values refer to a paired *t* test for a comparison of the ratios to 1.0, which would indicate the same bacterial count found by one of the automatic inoculators as found by manual pipette. *, *P* < 0.05.

c NS, nonsignificant.

Table 2 summarizes the comparative results obtained by the InoqulA-10 and WASP-10 instruments for 526 clinical urine specimens. The InoqulA-10 had more results with no growth (189 InoqulA-10 samples versus 141 WASP-10 samples) (*P* < 0.05), and the WASP-10 instrument had more results with commensals (108 WASP-10 samples versus 91 InoqulA-10 samples) (*P* < 0.005). The WASP-10 generally produced higher colony counts than those produced by the InoqulA-10. Specifically, excluding specimens for which both the InoqulA-10 and WASP-10 instruments produced commensals, mixed-growth, or no-growth results, there were 144 specimens with uropathogens with the same organism concentration ranges (e.g., 10^4^ to 10^5^ CFU/ml) for both instruments, 7 specimens for which the InoqulA-10 had higher organism concentrations than those produced by the WASP-10, and 105 specimens for which the WASP-10 had higher organism concentrations than those produced by the InoqulA-10 (*P* < 0.0001). These included 31 specimens for which the WASP-10 produced countable colonies and the InoqulA-10 produced no growth, and 5 specimens for which the InoqulA-10 produced countable colonies and the WASP-10 produced no growth.

**TABLE 2:** Comparison of results obtained with InoqulA-10 and WASP-10

InoqulA-10 results (no. of samples)	WASP-10 results (no. of samples)							
	No growth	Mixed growth	CFU/ml of:				Commensal flora only	Total
			10^2^–10^3^	10^3^–10^4^	10^4^–10^5^	≥10^5^		
No growth	133		27	3	1		25	189
Mixed growth^*a*^		19						19
CFU/ml								
10^2^–10^3^	5	2	53	13	8	3		84
10^3^–10^4^			1	10	21	4		36
10^4^–10^5^					5	25		30
≥10^5^					1	76		77
Commensal flora only	3		4			1	83	91
Total	141	21	85	26	36	109	108	526

a More than 2 urinary pathogens.

Table 3 summarizes the comparative results obtained by the InoqulA-10 and WASP-1 instruments for 526 clinical urine specimens. The InoqulA-10 had more results with no growth (189 InoqulA-10 samples versus145 WASP-1 samples; *P* < 0.005), and the WASP-1 had more results with commensals (113 WASP-1 samples versus 91 InoqulA-10 samples; *P* value was nonsignificant [NS]). The WASP-1 generally produced higher colony counts than those produced by InoqulA-10. Specifically, excluding specimens for which both InoqulA-10 and WASP-10 produced commensal, mixed-growth, or no-growth results, there were 90 specimens with uropathogens with the same organism concentration ranges (e.g., 10^3^ to 10^5^ CFU/ml) for both instruments, 6 specimens for which the InoqulA-10 had higher organism concentrations than those produced by the WASP-1, and 155 specimens for which the WASP-1 had higher organism concentrations than those produced by the InoqulA (*P* < 0.00001). These included 30 specimens for which the WASP-1 produced countable colonies and the InoqulA-10 produced no growth, and 5 specimens for which InoqulA-10 produced countable colonies and the WASP-1 produced no growth.

**TABLE 3:** Comparison of results obtained with InoqulA-10 and WASP-1

InoqulA-10 results (no. of samples)	WASP-1 results (no. of samples)						
	No growth	Mixed growth	CFU/ml			Commensal flora only	Total
			10^3^–10^4^	10^4^–10^5^	≥10^5^		
No growth	133		30			26	189
Mixed growth^*a*^		18				1	19
CFU/ml							
10^2^–10^3^	4	1	57	15	2	5	84
10^3^–10^4^	1		9	25	1		36
10^4^–10^5^				5	25		30
≥10^5^				1	76		77
Commensal flora only	7		2		1	81	91
Total	145	19	98	46	105	113	526

a More than 2 urinary pathogens.

**FIG 1:**
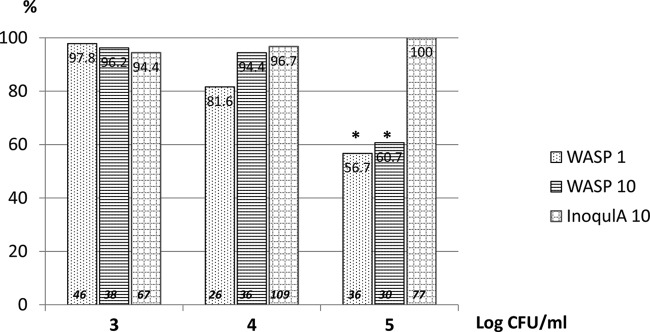
Percentage of cultures with ≥5 single colonies produced by WASP-1, WASP-10, and InoqulA-10 for cultures with 10^3^ to 10^4^, 10^4^ to 10^5^, and ≥10^5^ CFU/ml. The numbers at the top of the columns denote the percentage, and the numbers in italics at the bottom of the columns denote the number of samples tested for each category. *, *P* < 0.05.

**FIG 2:**
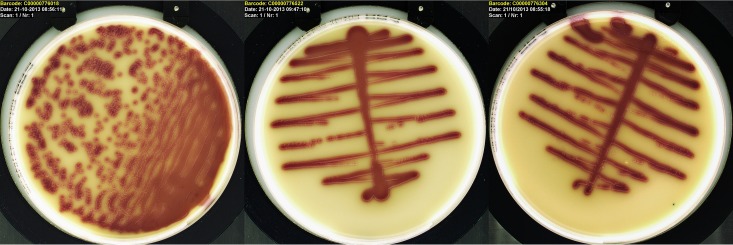
Pictures of a representative routine urine culture (E. coli on chromogenic agar [Brilliance UTI-agar; Oxoid]) inoculated with each of the three automatic inoculators, the InoqulA-10 (left), WASP-10 (center), and WASP-1 (right). The picture also illustrates the streaking patterns used in the study.

Using a definition of ≥5 isolated colonies as being generally acceptable for MALDI-TOF MS identification and antimicrobial susceptibility testing, the results were compared to determine how many times ≥5 isolated colonies of uropathogens were produced by the three inoculation methods (Fig. 1) For the WASP-1 and WASP-10 instruments (Fig. 1), the differences in the relative numbers of cultures with ≥5 isolated colonies decreased as the numbers of CFU increased in the cultures, while the differences in the relative numbers of cultures with ≥5 isolated colonies increased for the InoqulA-10 (significantly different for counts of ≥10^5^ [4] for InoqulA-10 compared to both the WASP-1 and WASP-10; Fig. 1). Figure 2 shows a representative chromogenic plate culture for the three inoculation methods for one of the urine samples in which there were ≥10^5^ CFU/ml of a uropathogen.

## DISCUSSION

For many microbiology specimens, results are evaluated in a nonquantitative (presence or absence of pathogens) or semiquantitative (few, moderate, or many) manner. However, for some specimen types, particularly urine specimens, the results are reported quantitatively and, indeed, culture workup and the potential clinical significance are predicated on the concentration of potential pathogens in the urine specimen (4–7). Consequently, it is important to assess the quantitative accuracy of plating urine specimens by automated instruments, such as the WASP and the InoqulA, as patient management decisions are driven by these culture results.

The results of our study using suspensions of quality control strains of E. coli and E. faecalis indicated that the InoqulA-10 and WASP-10 instruments produced results within 1 log_10_ of the results obtained with a calibrated pipette, with the mean of the WASP-10 results about 2-fold higher than that of the InoqulA-10 results. The WASP-1, however, produced results up 1 log_10_ higher than those obtained with a calibrated pipette.

The relative differences between the results obtained with the quality control organisms for the WASP and InoqulA instruments are likely due to the sampling methods used. Studies have indicated that the use of calibrated loops can be imprecise (3). Albers and Fletcher (8) noted that “the use of the calibrated loop by clinical microbiologists to determine colony counts from urine specimens is only semiquantitative and not very reliable, as the reproducibility of the method is dependent on many uncontrolled variables.” In an evaluation of the WASP instrument, Harrington and colleagues (9) noted that the colony counts produced by the WASP increased as the loops were inserted deeper into the specimen tube. This was most notable in this study with the WASP 1-μl loop.

We suspect that the relatively higher colony counts obtained with the WASP-1 and WASP-10 instruments compared to those with a calibrated pipette were due to the use of a wire loop to transfer the specimen from the transport tube to the agar plate. As noted by Albers and Fletcher (8), there are inherent inaccuracies associated with the use of a 1-μl loop. Importantly, the InoqulA and WASP instruments utilize different technologies to determine the depth of the liquid to which the transfer device (pipette versus loop) goes. The InoqulA instrument utilizes a liquid sensor technology to detect the specimen meniscus, after which the pipette tip is inserted 15 mm into the specimen (Paul Bourbeau, BD Diagnostics, personal communication). The WASP, on the other hand, inserts the loop to a fixed depth, as determined by programming for a specimen tube or specimen type. Importantly, the depth for the WASP loop is predicated on the minimum fill volume for a particular transport tube. For the tubes used in our study, the volume of urine varied from 4 to 10 ml.

In examining the relative colony counts obtained for clinical specimens between the InoqulA-10 and WASP-10 and between the InoqulA-10 and WASP-1, the results essentially paralleled the results obtained with the quality control organisms. For the InoqulA-10 versus WASP-10 analysis, the WASP-10 results overall were higher than the InoqulA-10 results.

In examining the number of isolated colonies produced by the WASP and InoqulA, the InoqulA produced a higher percentage of specimens with ≥5 isolated colonies than did either the WASP-1 or WASP-10. The differences were most pronounced for specimens with ≥10^5^ CFU/ml. The WASP-1 and WASP-10 produced 57% and 61% of the specimens, respectively, with ≥5 isolated colonies for specimens with ≥10^5^ CFU/ml, while the InoqulA-10 produced 100% of the specimens with ≥10^5^ CFU/ml. These differences can have significant economic and clinical implications. Croxatto and colleagues (10) estimated that at the University Hospital of Lausanne, Switzerland, the cost of reisolating an organism from a primary plate was 5.8 EUR per reisolation. Moreover, they noted that specimens requiring reisolation were delayed for an additional day before being reported.

There have been few studies that have assessed the quantitative accuracy of the WASP and InoqulA instruments. Bierma and colleagues (11) compared the performance of the WASP instrument with manual plating (manual plating method not specified) and noted that the WASP produced higher colony counts and/or additional species than those produced by the manual method. Bourbeau and Schwartz (12) reported similar colony counts obtained for urine specimens with the WASP and InocuLab instruments, but, importantly, the InocuLab utilizes a calibrated loop for specimen inoculation, similar to the WASP. Froment and colleagues (13) compared the performances of the InoqulA instrument and a manual loop method that included clinical urine specimens. They reported that higher colony counts were observed more frequently with the manual method than with the InoqulA instrument.

Our results indicate that the WASP produces higher colony counts than the InoqulA for urine specimens, particularly for WASP specimens plated with a 1-μl loop. Limited comparisons with a calibrated pipette suggest that the results produced by the InoqulA instrument are more accurate than the results obtained with the WASP instrument. In our opinion, the implications of these results are significant for the clinical laboratory, as quantitative urine culture results drive laboratory workup and reporting and subsequent patient management. We recommend that evaluations of quantitative plating of urine specimens utilizing automated instruments, such as the InoqulA and WASP, include a calibrated pipette for all specimens. Even after calibration, it is noteworthy that for a WASP 1-μl loop, the counts are so varied (large standard deviations, as noted in Table 1), it is impossible to count and report more reliably than ±1 log_10_ CFU/ml. We would recommend, as we routinely do in our laboratory, that all laboratories routinely inoculate at least 10 μl for a quantitative urine culture. Last, our results indicate that the InoqulA instrument produces more specimens with ≥5 isolated colonies than the WASP, particularly for specimens with ≥10^5^ CFU/ml.
